# Impact of Two Types of Exercise Interventions on Leptin and Omentin Concentrations and Indicators of Lipid and Carbohydrate Metabolism in Males with Metabolic Syndrome

**DOI:** 10.3390/jcm12082822

**Published:** 2023-04-12

**Authors:** Karol Makiel, Agnieszka Suder, Aneta Targosz, Marcin Maciejczyk, Agnieszka Kozioł-Kozakowska, Alon Haim

**Affiliations:** 1 Department of Anatomy, Faculty of Physical Rehabilitation, University of Physical Education, 31-571 Cracow, Poland; 2 Department of Physiology, Faculty of Medicine, Jagiellonian University Medical College, 31-531 Cracow, Poland; 3 Department of Physiology and Biochemistry, Faculty of Physical Education and Sport, University of Physical Education, 31-571 Cracow, Poland; 4 Department of Pediatrics, Gastroenterology and Nutrition, Faculty of Medicine, Jagiellonian University Medical College, Wielicka Str. 265, 30-663 Cracow, Poland; 5 Department of Pediatrics Endocrinology and Diabetes, Faculty of Health Sciences, Ben-Gurion University of the Negev, Beer-Sheva 653, Israel; 6 Soroka University Medical Center, Beer-Sheva 151, Israel

**Keywords:** omentin, leptin, exercise, metabolic syndrome, obesity, physical activity, QUICKI

## Abstract

Leptin (LEP) and omentin (OMEN) are proteins whose concentrations change with the development of the metabolic syndrome (MetS). There are few intervention studies using various forms of physical activity in people with MetS that aim to determine the impact of physical exercise on the fluctuations of the presented hormones, and their results are contradictory. The present study aimed to examine the effect of two types of exercise intervention on LEP and OMEN concentrations and indicators of lipid and carbohydrate metabolism in males with MetS. The study included 62 males with MetS (age 36.6 ± 6.9 years, body mass 110.31 ± 17.37 kg), randomly allocated to EG1, the examined group with aerobic training (*n* = 21); EG2, the examined group with combined aerobic and resistance training (*n* = 21), both for 12 weeks, and the control group (CG) without interventions (*n* = 20). Anthropometric measurements, body composition (body fat [BF], android body fat [ANDR]), as well as a biochemical blood analysis (omentin [OMEN], leptin [LEP], quantitative insulin sensitivity check index [QUICKI], high-density lipoprotein cholesterol [HDL-C] and nonHDL-C) were performed at baseline, and at 6 and 12 weeks of interventions and after 4 weeks after ending intervention (follow-up). Intergroup and intragroup comparisons were performed. In the intervention groups EG1 and EG2, a decrease in BF was observed as well as an improvement in carbohydrate metabolism parameters. In the EG1 group, the level of ANDR was reduced. In EG2 a decrease in LEP concentration between measurements was confirmed. However, no significant changes were found in the concentration of OMEN in any groups. Combined aerobic and resistance exercises led to a higher reduction of LEP concentration than applying only aerobic training in males with MetS.

## 1. Introduction

The metabolic syndrome (MetS) is diagnosed in patients who are affected by several mutually connected disorders leading to an increased risk of cardiovascular disease development, mainly atherosclerosis, insulin resistance and type 2 diabetes, and vascular and neurological complications such as cerebrovascular incidents [1]. Metabolic disorders become a syndrome if three out of five criteria are confirmed in a patient: abdominal obesity, low high-density lipoprotein cholesterol (HDL-C), high triglyceride levels, high glucose level, high blood pressure, or treatment for a specific disorder is conducted. The main, indirect cause of MetS is obesity which is always connected with the risk of type 2 diabetes, cancers and other chronic diseases that are components of the MetS [2,3]. Insulin resistance is thought to be the direct cause of diseases that the MetS consists of, mainly due to strong correlations with obesity and accompanying comorbidity [4]. Obesity favours the accumulation of visceral fat, which is connected with the occurrence of systemic inflammation of low intensity and adds to the development of metabolic disorders [5]. In a population of obese people at risk of the MetS, a dysregulated production of adipokines may lead to disorders in the functioning of insulin and glucose homeostasis [6]. Changes in adipokines levels, especially those produced in visceral fat tissue, may reflect systemic complications linked to obesity [7,8,9]. During the research overview it was found that white and brown adipose tissue communicate with skeletal muscles and the heart through the secretion of leptin (LEP) and omentin (OMEN) whose concentrations significantly alter with the development of obesity [10,11]. Concentrations of circulating biomarkers of inflammation condition, insulin resistance and LEP are significantly higher in people with MetS compared to the control group [12], whereas in the same population lowered levels of OMEN were observed [13]. 

Leptin (LEP) is an adipokine that due to its influence on the regulation of appetite and energy homeostasis attracts interest among researchers dealing with obesity and MetS [14]. Insulin and LEP act as the key signals regulating energy balance through direct influence on their related receptors in hypothalamus neurons affecting the control of food intake and energy expenditure [15]. Moreover, LEP plays many roles in the body, including a considerable role in immunology and respiratory systems, and it influences the regulation of sex hormones [16,17,18,19]. In obesity patients and in patients with MetS, the phenomenon of leptin resistance takes place [12,20]. The concentration of LEP level in serum is proportional to the level of obesity and it reflects the energy state of the body. The BMI threshold when the concentration of LEP starts increasing is 24.6 kg/m^2^ [11]. Leptin resistance is the main factor that leads to a progression of the MetS and understanding of the mechanism of leptin resistance development requires further research [4]. An improper level of LEP may result in the development of type 2 diabetes (T2DM) [21], cardiovascular diseases [22] and some cancers [23,24]. 

Omentin (OMEN) occurs in two forms: OMEN-1 and OMEN-2 [25]. OMEN-1 is the main protein among isoforms that exists in the human body [13,26]. Unlike most adipokines, OMEN is not produced in subcutaneous fat tissue [27]. It is mainly synthesized in visceral fat tissue [25]. Despite this, its lowered concentrations are observed in a population of obese people and its decrease leads to metabolic disorders such as insulin resistance and glucose intolerance. People with proper body weight feature a much higher concentration of OMEN in serum than those overweight [13]. There are also differences in OMEN concentrations in the case of different sex—women have higher concentrations of OMEN than men [13]. The level of OMEN may allow us to predict metabolic consequences or diseases co-existing with obesity [13]. In relation to other adipokines, it was demonstrated that OMEN is positively connected with the level of adiponectin in serum and negatively with levels of LEP [13]. Low concentrations of OMEN in serum are also found in patients with type 1 and type 2 diabetes [28,29]. Secretion of OMEN-1 is stimulated in response to applying physical activity. Physiological adaptation of skeletal muscles to physical activity can also be related to the action of OMEN [30]. Conducting intervention research with the use of aerobic or resistance training leading to the reduction of adipose tissue offers opportunities for a better understanding of how the discussed adipokine works. 

Regular physical activity is an important element of a lifestyle that prevents metabolic complications [31]. The American College of Sports Medicine recommends introducing 200–300 min of moderate physical activity weekly for obese and overweight people in order to achieve a clinically significant reduction of body mass [32]. The researchers highlight benefits resulting from applying aerobic-resistance training as an element of intervention in health condition improvement in people with a surplus of adipose tissue. An important area of the positive influence of resistance training on health conditions in people with surplus body mass comprises beneficial hormonal changes in the levels of adiponectin, leptin and insulin and improvement of MetS parameters [33]. Resistance training does not always lead to body mass loss, but it can increase fat-free mass and decrease adipose tissue, and changes in proportion in the presented tissues are connected with the improvement of health conditions, reducing, among others, the level of insulin resistance, inflammation and atherogenic lipids [34].

A better understanding of roles played by LEP and OMEN in MetS may help choose more precise therapeutic interventions by the selection of proper training methods leading to maximum effects from attempts to reduce body mass and improve health [35,36,37]. Modification of lifestyle by introducing physical activity may lower insulin resistance. However, test results do not indicate clear results connected with changes in adipokine concentrations influenced by physical activity in patients with MetS so the authors suggest that more research should be conducted in the field [38]. 

The aim of the project was to evaluate the influence of applying two types of physical training for 12 weeks (aerobic vs. combined aerobic-resistance) and four weeks of follow-up on the concentration of LEP and OMEN and changes in indicators of lipid and carbohydrate metabolism in males with MetS. We hypothesize that combined aerobic-resistance training through the development of muscle mass and loss of adipose tissue brings more beneficial changes in concentrations of LEP and OMEN than aerobic training. 

## 2. Materials and Methods

### 2.1. Materials

The research is part of a project whose methodology has already been described in the paper presenting the effects of exercise interventions on the concentration of irisin and interleukin-6 [39]. Below, the description of the research methodology was supplemented with elements related to the present paper.

The research, planned as a prospective, randomized, and controlled trial study, was to examine the results of applying two kinds of physical training for 12 weeks (aerobic vs. combined aerobic-resistance) in male subjects with metabolic syndrome (MetS). The study examined the effects on their body composition, changes in leptin (LEP) and omentin (OMEN) levels as well as indexes of the MetS in comparison to men with MetS, contained in the control group, who did not take part in the interventions. After the training period finished, the subjects from all the groups were monitored for the subsequent 4 weeks as a follow-up phase.

**Figure 1:**
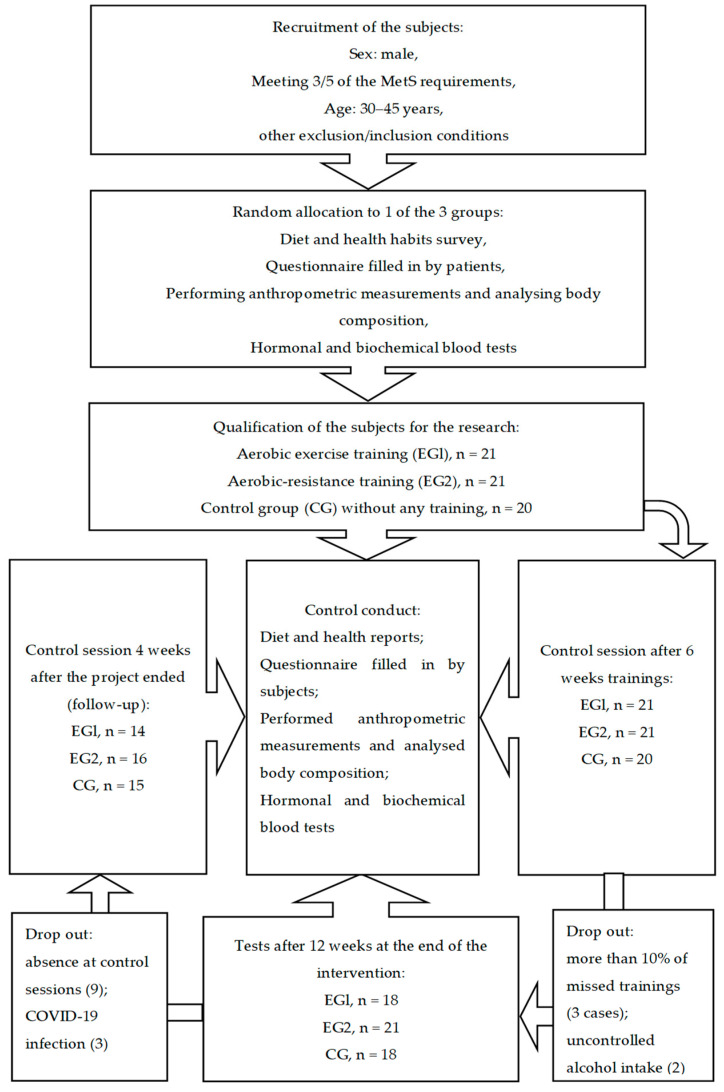
The course of the study.

In order to prevent bias, the laboratory staff, statisticians and analysts were not informed about the subjects’ group allocation, but because of the type of intervention, we did not employ the blind trial. The research was registered in the clinical trials registry on the ANZCTR platform (Australian New Zealand Clinical Trials Registry): ACTRN 12622001394730 and received the approval of the Ethics Committee of the Regional Medical Chamber in Cracow (90/KBL/OK/2020). Figure 1 presents the study course.

The study involved 62 men aged 30–45 (mean age 37 ± 7) years with elevated waist circumference (WC) ≥ 94 cm and with 2 out of the 4 MetS criteria recognised: hypertriglyceridemia under treatment or concentration of triglycerides > 150 mg/dl (1.7 mmol/l); concentration of HDL-C < 40 mg/dl (1.03 mmol/l)—in men or the lipid disorder under treatment; systolic blood pressure (SBP) ≥ 130 mm Hg or diastolic (DBP) ≥ 85 mm Hg, or treatment of previously diagnosed hypertension; fasting level of GL in blood plasma ≥ 100 mg/dl (5.6 mmol/l) or pharmacological treatment of diabetes type 2 (IDF, International Diabetes Federation, 2006 [40]). The exclusion criteria involved a lack of medical statements concerning no contraindications to undertake aerobic-resistance exercise and others described in the former article in the field [39]. 

The subjects were allocated randomly into 3 groups; the assignment relied on simple randomization following the sealed opaque envelopes:

the experimental group (EG1) of men with MetS (*n* = 21) realizing aerobic training (age: 34.21 ± 6.06; body mass index, BMI: 34.57 ± 4.58; waist circumference, WC: 114.7 ± 10.93; waist to height ratio, WHtR: 63.37 ± 6.22);
the experimental group (EG2) of men with MetS (*n* = 21) realizing combined aerobic-resistance training (age: 37.37 ± 7.08; BMI: 33.14 ± 4.32; WC: 114.8 ± 11.64; WHtR: 63.90 ± 5.97);
the control group (CG) of men with MetS (*n* = 20) not realizing any training (age: 38.26 ± 7.43; BMI: 33.20 ± 4.31; WC: 115.3 ± 10.54; WHtR: 63.72 ± 4.99). There were no differences between age and basic anthropological parameters before the interventions.

The subjects received detailed information about the procedures and aim of the study and about the option to give up the interventions at any stage. There were cases of patients resigning from the training and of exclusion of participants due to: more than 10% of missed training (3 cases), uncontrolled alcohol intake (2), absence at control sessions (9), and COVID-19 infection (3). The subjects declared not to change their diet, administered remedies, and spare time activity while participating in the research. They all submitted written consent to take part in the project and accepted using their data and the study results for academic aims. 

### 2.2. Methods

The assessments found below were conducted in all the subjects and took place four times: before the training started, during the project (after 6 weeks and after 12 weeks of training) and 4 weeks after the end of the training sessions (follow-up):

#### 2.2.1. Anthropometry

Body height (BH) [cm], body mass (BM) [kg] and waist circumference (WC) [cm] were measured for the needs of the study. BH was taken without shoes, in a standing position to the nearest 1 mm, with the head in the Frankfurt plane, with a stadiometer (Seca 231 stadiometer, Hamburg, Germany). BM was measured in the standing position with a standardized medical scale (Beurer PS 240, Budapest, Hungary), with an accuracy of 50 g. Waist circumference (WC) was taken to the nearest 1 mm with an anthropometric tape between the lower edge of the costal arch and the upper edge of the iliac crest with the subject in a standing position and registered at the end of a gentle expiration. Waist-to-height ratio (WHtR) was obtained by dividing waist circumference (in cm) by height (in cm).

#### 2.2.2. Body Composition

Body fat (BF) [kg], android body fat (ANDR) [%] and body mass index (BMI) [kg/m^2^] were assessed with the use of Dual-Energy X-ray Absorptiometry (DEXA). Measurements were done with the Lunar Prodigy Primo PR+352163 (Chicago, IL, USA) device following the manufacturer’s manual.

#### 2.2.3. Hormonal Blood Indexes

Samples of blood were taken in the morning after a 12-h fast and after a 24-h break from training, from the basilic, cephalic, or median cubital vein into test tubes (Vacumed^®^ system, F.L. Medical, Torreglia, Italy) by experienced nurses. They were then immediately centrifuged (RCF 1000× *g*) for 15 min at 4 °C (MPW-351R, MPW Med. Instruments, Warsaw, Poland) and plasma was collected and stored at −80 °C until further study (BIO Memory 690L, Froilabo, Paris, France). Leptin and omentin concentrations were assessed with ELISA kits according to the manufacturer’s guidelines. The human Leptin Sandwich ELISA Kit (catalogue number EIA-2395) was provided by DRG Instruments GmbH (Marburg, Germany). The human omentin ELISA kit (catalogue number 201-12-0156) was bought from Shanghai Sunred Biological Technology Co. (Shanghai, China). An ELx 808 spectrophotometric microplate reader (BioTek, Winooski, VT, USA) was applied to specify the optical density at 450 nm. Marking was performed in the Laboratory of Genetics and Molecular Biology at the Department of Physiology, Jagiellonian University Medical College, Cracow, Poland.

#### 2.2.4. Biochemical Blood Indexes

Sensitivity to insulin was determined applying the quantitative insulin sensitivity check index (QUICKI) [41], following the formula:

QUICKI = 1/[ log INS (µIU/ml) + log GL (mmol/l)]

Plasma insulin (INS) [µIU/ml] concentration was assessed using electrochemiluminescence (ECLIA) with the Cobas e801 apparatus (Roche Diagnostics International Ltd., Mannheim, Germany). Glucose (GL) [mmol/l] concentration in the blood plasma was conducted by the enzymatic method with the Cobas c701/702 biochemical analyser (Roche Diagnostics International Ltd., Mannheim, Germany). The assessments were realized in conformity with the manufacturer protocol using reagents dedicated to the GLUC3 and Elecsys Insulin analysers, respectively.

The plasma levels of total cholesterol (TC) and high-density lipoprotein cholesterol (HDL-C) were specified with the spectrophotometric method relying on guidelines of the clinical chemistry analyser Architect ci-4100 (Abbott Laboratories). The intra- and interassay coefficients of variation (CV) for the assays were 0.9–1.2 and 1.2–1.8%, respectively. Non-HDL cholesterol (nonHDL-C) fraction was determined following the formula:

nonHDL-C [mmol/l − 1] = TC [mmol/l − 1] − HDL-C [mmol/l − 1]

#### 2.2.5. Evaluation of Energy Expenditure and Energy Value of Diet 

The International Physical Activity Questionnaire (IPAQ) was performed to estimate total physical activity. The calculation of the total estimation of activity relied on the MET (metabolic equivalent of a task) formula expressed as a value of for example 3 MET (intensity of slow cycling) × 30 min (duration per day) × 2 times per week (frequency) = 3 [MET] × 30 [min] × 2 [week] [MET min/week] and based on the sum of non-exercise activity thermogenesis (NEAT) assessed with the use of the IPAQ and energy expenditures connected with training sessions in EG1 and EG2 [42].

A quantitative assessment of the diet and the monitoring of changes in it while taking part in the project were realised based on the 24 h interview with nutrition record that was conducted by an experienced nutritionist. The results were elaborated in the DietaPro program (version 4.0, Institute of Food and Nutrition, Warsaw, Poland). The energy value of the diet was calculated as [kJ/day].

### 2.3. Exercise Interventions

Monitoring of intensity in both forms of interventions of aerobic character as well as the amount of load in resistance intervention were detrained individually, according to guidelines of the American College of Sports Medicine [43]. Following the project assumptions, the subjects realized three training sessions weekly that were converted into 3 × 6 MET of energy expenditure per week for aerobic training and 3 × 5.5 MET for the resistance one [42]. The physical activity interventions took place in a fitness club in Cracow. Each training was supervised by the same personal coach, at the same daytime (6–9 pm), at the same temperature (22 Centigrade) and humidity. The subjects’ presence at the training sessions was registered and consequently, participants were eliminated from observation and statistics due to their absenteeism being higher than 10% during the 12-week course of training. 

Before the resistance training, the test of 1 repetition maximum (1RM) was determined. The subjects took the 1 RM test before the intervention and after 6, 12 and 16 weeks. The obtained load and number of repetitions were converted into 1 RM based on a 1 RM calculator [44]. The exact course of the 1RM test is described in the Supplement.

#### 2.3.1. Aerobic Training

The aerobic training sessions (Figure S1, Supplement) were performed in max 5-person groups, 3 times a week, starting with a 5-min warm-up, not exceeding 50% maximal heart rate (HR max). After they warmed up, the intensity was elevated to 70% HR max due to higher treadmill speed or angle (treadmill walk, Technogym New Excite Run Now 500, Cesena, Italy), resistance on upright bikes (Technogym Artis, Cesena, Italy) and movement range or resistance on X-trainer (Precor EFX556i Elipsa, Woodinville, WA, USA). The subjects could use these three devices alternately. HR was monitored by a running watch Polar M200 GPS with a wrist HR sensor (Kempele, Finland). The aerobic training duration was 45 min. It was continuous with constant HR. The stretching phase of the engaged muscle groups lasted 10 min. 

#### 2.3.2. Combined Aerobic-Resistance Training

The aerobic-resistance training (Table S1, Supplement) was performed in max 5-person groups, 3 times a week. Initially, a 5-min aerobic warm-up in the form of a treadmill walk was applied (Technogym New Excite Run Now 500, Cesena, Italy), to reach 50% HR max. In the first phase of resistance training, 3 complex exercises engaging the whole body (FBW—full body workout) were applied in 4 series with 120 s breaks between them. In the second week 3 series with 6 exercises each and 90 s breaks were introduced. Starting with the third week, the training included 3 series with 9 exercises each and 60 s breaks. 

The load was initially set at 50% of 1 RM and after 4 weeks it was increased to 70%. After resistance exercises, there was an aerobic training element: the participants trained on a treadmill (Technogym New Excite Run Now 500, Cesena, Italy), upright bike (Technogym Artis, Cesena, Italy) or x-trainer (Precor EFX556i Elipsa, Woodinville, WA, USA) with an intensity of 50% HR max in the first week and 70% HR max from the second week of intervention. Duration of the resistance training was respectively 30, 35 and 40 min (1st, 2nd and 3rd week), and then respectively 20, 15 and 10 min for the aerobic one. The stretching phase was 5 min. The duration of the aerobic-resistance intervention was 60 min. Progression of load (kg) in selected resistance exercises calculated based on 1 RM [45] for EG2 was statistically significant in the analysed period (Table S2, Supplement).

### 2.4. Statistical Analysis

Statistical significance concerning the number of subjects relied on previous studies in the area found in the literature. Calculation of the sample size applied an error probability (α) of 0.05, power (1 − β) of 0.80, and an average effect size (d) of 0.8 and for the tested sample was *n* = 54.

The Shapiro-Wilk test was used to check the distribution of the results for the analysed variables. Because of a normal distribution of most variables, the differences between the examined groups and the control one were assessed with the one-way analysis of variance for independent groups. A comparison of the intervention influence on changes in the analysed variables between EG and CG was performed with the ANOVA test for dependent groups with post-hoc comparison (Tukey test). The size effect (ES) was assessed for the ANOVA test:

$$\eta^{2}=\frac{{SS}_{effect}}{{SS}_{total}}$$

where squared eta (η) is the ratio of the sum of squares (*SS*) for the effect divided by the total sum of squares (*SS*). Squared eta applies interpretation guidelines by Cohen 0.1 ≤ 0.3 (low effect), 0.3 ≤ 0.5 (moderate effect) and ≥0.5 (high effect) [46]. Pearson correlation coefficient (r) was calculated. 

Multiple regression was used to explain the variation in LEP levels. The model was prepared with the use of the econometric linear multiple regression model assessed by the least squares method. In the model, the residual standard errors and test *p*-values were corrected using robust standard errors corrected for heteroscedasticity. 

In all analyses, effects were considered significant if their probability value *p* was lower than the assumed significance level α = 0.05 (*p* < 0.05). The ggplot2 package of the RStudio IDE in the R programming language was used to perform all calculations.

## 3. Results

After applying health training intervention both in EG1 (*p* = 0.04) and EG2 (*p* < 0.001) a significant increase of MET [min/week] was confirmed between the initial measurements and measurements in 6th (*p* < 0.001) and 12th (*p* < 0.001) weeks of intervention (Table 1). In follow-up, an increase of MET was also confirmed in EG1 (*p* = 0.03) and EG2 (*p* = 0.04). No significant changes were found in MET in CG. The intergroup analyses confirmed differences (*p* < 0.001) in the 6th week of observation between EG1 and CG (*p* < 0.001), and EG2 and CG (*p* = 0.04) (Table 1). 

The energy value of the diet [kJ] during the intervention after 6 (*p* = 0.68), and 12 (*p* = 0.24) weeks did not change in EG1. In follow-up, an increase in calorie intake was registered (*p* = 0.01). In EG2 a gradual increase of consumed calories in the diet between measurements was observed (*p* < 0.001). In CG, the energy value of the diet during the observation period did not change significantly (*p* = 0.12) (Table 1).

After the intervention in EG1, there was a decrease in body mass (*p* < 0.001) between measurements, the largest reduction in body mass occurred after 6 weeks of intervention −2.6 kg (*p* < 0.001) (Table 2). There were no significant changes in body mass in the EG2 and CG groups. 

A decrease in BF [kg] in EG1 (*p* = 0.01) and in EG2 (*p* < 0.001) was confirmed (Table 2). The largest decrease in BF (average −1.47 kg) took place in EG1 after 6 weeks of intervention (*p* < 0.001) and in EG2 (average reduction of −2.2 kg) after 12 weeks of intervention (*p* = 0.01). No significant changes were observed in the level of adipose tissue in CG. 

The decrease in the percentage of visceral fat (ANDR) [%] was confirmed in EG1 (*p* = 0.04), with the largest changes occurring after 12 weeks of intervention; an average decrease of −3.4% ANDR (*p* = 0.03). In EG2, a decrease in ANDR was also observed, but these changes were not significant. Statistically significant changes in ANDR were not confirmed in the CG group (Table 2). 

The applied intervention with aerobic physical activity increased the value of QUICKI (*p* = 0.02) in EG1 (Table 3). In EG2, an initial decrease in QUICKY after 6 weeks of intervention (*p* = 0.04) was confirmed, followed by an increase in QUICKI (*p* < 0.001) between measurements. No significant changes in CG were observed. 

No changes in nonHDL-C levels were confirmed in any of the groups as a response to the intervention (Table 3). After 6 weeks of intervention, a decrease in HDL-C concentration in EG1 was observed, followed by an increase above baseline between weeks 6 and 16 of the study (*p* = 0.04) (data not included). In the EG2 group, a gradual increase in the mean HDL-C value was observed, but it was not statistically significant. Changes in HDL-C concentration in CG were insignificant (Table 3).

**Figure 2:**
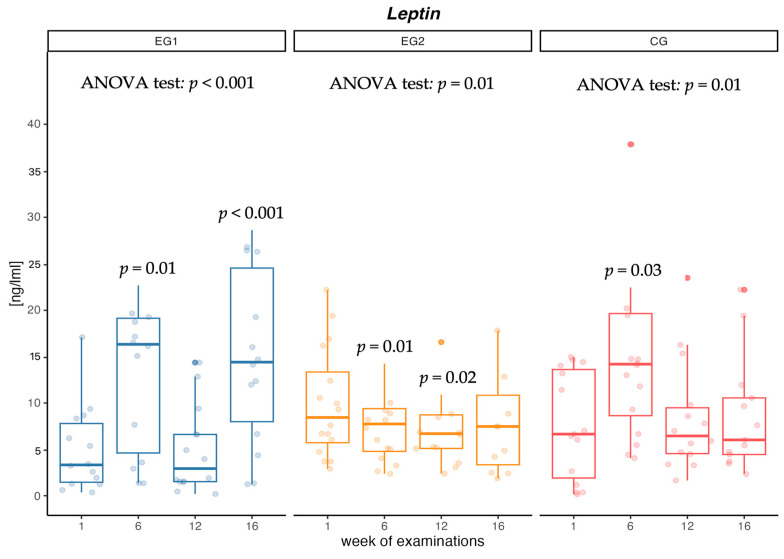
Changes in leptin (LEP) concentration [ng/ml] in aerobic group (EG1), aerobic-resistance group (EG2) and control group (CG) during weeks of examinations.

The obtained results indicate changes in LEP concentration between measurements in all observed groups (Table 4, Figure 2). In EG1, after an initial increase (*p* = 0.01), a significant decrease was confirmed at week 12 (*p* = 0.01) of the intervention. In the follow-up period, the LEP concentration increased again (*p* = 0.01). In EG2, a gradual decrease in LEP concentration was observed during the intervention period—after 12 weeks, LEP concentration was reduced by an average of −2.53 ng/ml (*p* = 0.02). In the follow-up period, a slight increase in LEP concentration was also observed (*p* = 0.05). In the CG group, there was a significant increase (*p* = 0.03) in LEP concentration after 6 weeks of observation. Differences between the EG2 and CG groups (*p* = 0.03) were confirmed in the 6th week of observation and in the follow-up period between EG1 and EG2 (*p* = 0.03). 

No changes in OMEN concentration were observed both between groups and between measurements. Despite the lack of statistically significant changes, it was shown that in the intervention groups (EG1 and EG2) the level of OMEN increased after 6 and 12 weeks in relation to the initial values (Table 4).

Significant correlations (Table 5) were confirmed in EG1 between LEP and MET (r = −0.37), the energy value of diet (r = 0.28), BM (r = 0.28), BF (r = 39), ANDR (r = 0.39), QUCIKI (r = −0.45) and for cholesterol fraction nonHDL-C (r = 0.50) and HDL-C (r = −0.43). No significant correlations were found in EG1 for OMEN. In addition, significant correlations were confirmed in EG1 between QUCIKI and MET (r = 0.29), the energy value of diet (r = −0.35), HDL-C (r = 0.72) and components of body composition: BM (r = −0.67), BF (r = −0.61), ANDR (r = −0.56). In the EG2 group, very strong correlations were confirmed between LEP and components of body composition: BM (0.73), BF (r = 0.88), ANDR (r = 0.87), and the QUICKI carbohydrate metabolism index (r = −0.45). In the EG2 group, a negative correlation between OMEN and nonHDL-C was confirmed (r = −0.39). As in the EG1 group, in the EG2 group there were strong correlations between QUICKI and components of body composition: BM (r = −0.64), BF (r = −0.55), ANDR (r = −0.44). In CG, LEP level was associated with BF (r = 0.51), ANDR (r = −0.47), and BM (r = 0.42). In the case of OMEN, correlations were confirmed for BM (r = −0.39) and HDL-C (r = 0.31). Correlations in CG for QUICKI occurred between BM (r = −0.41), BF (r = −0.33) and HDL-C (r = 0.28).

The applied multiple regression model showed a significant connection of BF with the concentration of LEP (*p* < 0.05). The variability of LEP was explained by the analysed variables in 10% (value of R^2^ model = 0.10) (Table 6).

## 4. Discussion

The aim of the study was to assess the influence of two types of physical training (aerobic vs. combined aerobic-resistance) realised for 12 weeks in a group of males with MetS on changes in body composition, concentrations of leptin (LEP) and omentin (OMEN), and MetS indexes in comparison with the control group. The research results confirmed significant changes occurring in the level of LEP under the influence of the applied exercise interventions, but also in the control group fluctuations of the hormone were observed. The obtained results indicate a significant correlation between LEP and the place of its synthesis, i.e., adipose tissue, including visceral fat and the change taking place in the fat tissue during the interventions. The favourable influence of aerobic and combined aerobic-resistance training was also confirmed on the insulin resistance index QUICKI whose changes were significantly related to changes occurring in LEP. The required level of significance needed to confirm changes in the levels of OMEN, HDL-C and nonHDL-C was not achieved. 

The present study confirmed the variability in LEP concentration between measurements in all groups, however, the biological behaviour of the hormone differed in each of the analysed groups. After 6 weeks of intervention, there was an increase in LEP concentration in the aerobic group, but the extension of the intervention time to 12 weeks was associated with a downward trend in LEP concentration in our study. In a meta-analysis Yu et al. presented several reports that aerobic exercise has a significant effect on lowering serum LEP levels [47]. In the study by Klempel et al. [48], it was shown that even a small weight loss (4–5%) can have a beneficial effect on LEP concentration. In the case of a weight loss of 2.4%, there was a decrease in serum LEP concentration [49]. In our study, despite the initial weight loss, the LEP concentration increased in the EG1 group after 6 weeks of intervention. The increase in LEP levels despite the use of aerobic training could be the result of other factors beyond our control. The study was conducted during the COVID-19 pandemic and, among others, increased anxiety levels due to the ongoing lockdown [50], less sleep [51], and increased stress levels [52] are factors connected with the fluctuation of hormones [53].

However, in the group with the aerobic-resistance intervention, a decrease in LEP concentration was confirmed both after 6 and 12 weeks of the intervention. LEP values in the EG2 group were significantly lower after 6 weeks compared to the CG group and after 16 weeks compared to the EG1 group. Due to the observed decreases in the LEP value in the EG2 group between measurements and differences between the groups, our results indicate a more beneficial effect of aerobic-resistance training than aerobics alone or no physical activity on the level of LEP. Such changes may relate to inflammation reduction in response to resistance-aerobic training [39]. Different results were obtained by the authors in the population study of people with MetS, in which Nordic Walking (NW) training at the level of 65–75% HR max or resistance training for a period of 12 weeks was used. Researchers did not confirm a change in LEP concentration in people practising resistance training, but they confirmed a decrease in LEP level by 27% in a group using NW [49]. Such direction of changes can result from the lack of changes in fat free mass (FFM) in any of the groups and a higher reduction of fat tissue in the NW group. Other studies have shown that LEP concentration decreased by 21% during a 3-month dynamic resistance training program [53] and by 14% during a 6-month program combining diet with moderate activity [54]. The scientists found out that LEP ameliorates the level of mRNA peroxisome proliferator-activated receptor gamma co-activator 1 (PGC-1) in the skeletal muscles and exercise of higher intensity can influence the adaptive decrease of LEP level [55,56].

In our research, an increase in the level of LEP concentration in CG was registered, which may be associated with the development of leptin resistance in individual people with MetS, not undertaking physical activity. 

Our study showed positive correlations between LEP concentration and BF in each group, which confirms the reports that serum LEP concentration depends mainly on adipose tissue mass [57]. Despite reports that subcutaneous adipocytes secrete more LEP than visceral adipocytes [58], our study confirmed positive correlations between LEP and the level of ANDR in each group. Correlation values between LEP and BF and ANDR are very similar, which may indicate a similar level of LEP synthesis in subcutaneous and visceral adipose tissue in the population of people with MetS. 

Negative correlations between LEP and QUICKI and statistical variability in both groups in the case of LEP and QUICKI confirm a highly probable cause-and-effect relationship of the effect of LEP on the level of insulin resistance in people with MetS. A similar correlation between LEP and QUICKI in MetS was confirmed by other researchers [59].

The obtained results confirm a strict relationship between LEP and the level of total and visceral adipose tissues which are the place of its synthesis [60]. The process of leptin resistance, occurring in obesity, plays the key role in complications connected with its course [61]. The results of our research confirm a high concentration of LEP in the population of people with MetS and obesity as well as a correlation between LEP and QUICKI. The therapeutic potential resulting from applying aerobic training and the combination of resistance and aerobic training, offers perspectives in treating and preventing MetS and obesity through influencing the reduction of the adipose tissue level and, consequently, a decrease of LEP level and insulin resistance.

The results of our work indicate a growing trend of mean concentrations of omentin (OMEN) in subsequent weeks of the aerobic intervention, however, the changes in OMEN values did not reach statistical significance. In the study of de Souza Batista et al. the authors emphasize the influence of exercise intervention in increasing OMEN concentrations [13]. An increase in the concentration of circulating omentin was also observed in people who underwent a 12-week aerobic training and whose level of fat content in the body decreased [62]. 

The main site of OMEN synthesis is visceral fat [25], therefore, in order to understand the variability of the presented hormone, we used the results estimated by the DEXA method. Analysing the level of visceral fat in the group subjected to aerobic intervention, its gradual decrease of 3.8% over 16 weeks was confirmed. In the group with aerobic-resistance intervention, its decrease in the same period by 3.3% was also confirmed, but it was not a significant change. Despite reports of a negative correlation between the level of OMEN and BMI, waist circumference and the level of visceral fat [13], our study did not confirm the correlation between the level of OMEN and the level of total and visceral fat in any of the groups. A possible reason for not achieving a significant increase in the OMEN concentration in the intervention groups may be insufficient loss of visceral fat. Weight loss is considered a key factor in an intervention to reduce pro-inflammatory cytokine levels and increase anti-inflammatory cytokine levels [63,64]. Achieving greater weight loss in individuals with MetS could result in clinically beneficial changes in OMEN concentrations. Similar relationships were confirmed by researchers in the case of other adipokines. Among patients achieving a weight loss of 4–5%, no significant changes in the concentrations of adiponectin, IL-6 and RBP4 were confirmed, while the concentration of LEP decreased [48]. The effect of lowering the OMEN concentration was achieved, among others, in a study with dietary intervention, where a low-energy diet (deficit of 500–1000 kcal per day) was used for 4 months. As a result of the intervention, a reduction in body weight of 13.8% was obtained. Researchers confirmed a decrease in LEP concentration by 60.6% and a significant increase in OMEN concentration by 22.1% [65]. 

Our study did not confirm the correlation between OMEN and LEP concentration and insulin resistance indices. The positive relationship between HDL-C and the level of OMEN was confirmed in CG. Despite the fact that OMEN is considered an adipokine whose important function is to influence the level of insulin resistance [18,30], no correlation between omentin and QUICKI was confirmed in any of the analysed groups. The significant correlations between visceral and total adipose tissue and the index of insulin resistance can be explained by the fact that the disproportionate accumulation of fat in the abdominal region is associated with reduced insulin-mediated glucose transport. Increased pancreatic beta cell apoptosis, necrosis or autophagy may be involved in beta cell dysfunction in MetS [66].

The results of our study indicate that HDL-C level increased by 5.8% in the aerobic intervention group and by 8.3% in the aerobic-resistance group over 16 weeks, although the changes were not significant. In the meta-analysis of studies analysing the impact of physical activity on lipid parameters, it was confirmed that the use of a combination of resistance and aerobic training over 12 weeks led to an increase in HDL-C from 3.5% to 23% [67]. The meta-analysis of studies on the subject of the lipid profile in people using resistance training also showed beneficial changes in nonHDL-C and HDL-C in people with the intervention [68]. Despite numerous reports on the beneficial effect of physical exercise on the level of nonHDL-C, no significant beneficial changes in the described parameter were observed in our studies. An increase in HDL-C concentration and a decrease in nonHDL-C concentration have been confirmed in other intervention studies using physical activity in the treatment of MetS [69]. Undertaking resistance training can lead to a 6% decrease in nonHDL-C levels and an increase in HDL-C levels by 1%. In the case of aerobic training, a decrease in nonHDL-C by 2.5% and an increase by 4% in HDL-C were confirmed [70]. No changes in HDL-C and nonHDL-C levels despite the intervention could result from the proportion of fatty acids supplied in the diet [71].

The study is not free from some limitations. The presented results show changes in LEP, OMEN concentrations and biochemical parameters under the influence of intervention in the form of physical activity, however, people participating in the study may have been exposed to other factors, such as increased stress levels, a limited amount of sleep, etc. that may affect the level of the presented parameters. Due to the long period of the study, the initial number of participants was reduced, which could have resulted in the lack of statistical significance in some measured parameters. During the follow-up procedure, the VO2 max test was not performed, which would allow monitoring of training adaptation in the intervention groups. Despite the initial assumptions of maintaining the current diet and constant control of the diet of the study participants, the amount of energy supplied in food increased. Describing the diet, no detailed analysis of fatty acids that could affect the lipid profile of the study participants was made.

To summarise, the use of a combination of resistance and aerobic training in men with MetS leads to a decrease in LEP concentration. Obtaining results is more beneficial when using a combination of two forms of exercise compared to using aerobic training alone. Taking up physical activity did not lead to significant changes in the concentration of OMEN in men with MetS. The use of aerobic training as well as a combination of aerobic and resistance training results in beneficial changes in the level of insulin resistance and body composition. However, a combination of aerobic and resistance training led to greater benefits in reducing the level of BF, BM, and ANDR. Leptin is an important adipokine whose significant relations with body composition and the level of insulin resistance in the population of males with MetS indicate that application of physical training in order to improve health conditions features a therapeutic potential. The use of a combination of aerobic and resistance training had a direct impact on the decrease in leptin levels during the 12-week intervention period. Explaining the role of OMEN in the process of MetS treatment support through introducing physical exercise requires a higher number of tests.

## Figures and Tables

**Table 1:** Metabolic equivalent of task (MET) and energy value of the research participants’ diet in the aerobic group (EG1), aerobic-resistance group (EG2), and control group (CG).

	Group	Week 1 Baseline	Week 6 Intervention	Week 12 Intervention	Week 16 Follow up	*p*-Value			
		$\bar{X}$ ± SD	$\bar{X}$ ± SD	$\bar{X}$ ± SD	$\bar{X}$ ± SD	Test ANOVA (ES)	d 6-1 (ES)	d 12-1 (ES)	d 16-1 (ES)
MET [min/week]	EG1	2214.40 ± 681.75	3127.22 ± 578.50	3134.06 ± 639.22	3204.26 ± 1507.68	0.04 (0.17)	<0.001 (−5.09)	<0.001 (−1.99)	0.03 (−0.66)
	EG2	2225.22 ± 522.06	2899.62 ± 412.91	3246.25 ± 1726.13	3264.50 ± 1740.83	<0.001 (0.12)	<0.001 (−2.18)	<0.001 (−0.63)	0.04 (−0.57)
	CG	2423.69 ± 705.72	2379.42 ± 693.42	2428.00 ± 674.38	2533.33 ± 724.75	0.67 (0.00)	0.70 (0.79)	0.84 (0.85)	0.23 (0.23)
	*p*-value	0.60	0.00 *	0.13	0.34				
Energy value of diet [kJ/day]	EG1	11,286.50 ± 1376.01	12,124.84 ± 1386.59	12,184.03 ± 1701.68	12,203.01 ± 1858.68	0.02 (0.55)	0.68 (0.13)	0.24 (−0.33)	0.01 (−0.77)
	EG2	10,732.99 ± 872.50	11,486.72 ± 1194.02	11,671.27 ± 1760.909	11,867.81 ± 1706.025	<0.001 (0.06)	0.02 (−0.77)	<0.001 (−1.13)	<0.001 (−1.56)
	CG	11,158.80 ± 1565.82	11,248.34 ± 1444.98	11,462.44 ± 1372.21	11,695.18 ± 1442.73	0.12 (0.05)	0.90 (−0.04)	0.38 (−0.26)	0.05 (−0.65)
	*p*-value	0.79	0.57	0.92	0.97				

* post-hoc: EG1-CG: *p* < 0.001, EG2-CG: *p* = 0.04; d 6-1, d 12-1, d 16-1—differences in results after 6 and 12 weeks of training, respectively, and after 4 weeks of follow-up compared to measurements obtained before training, $\bar{X}$—mean, SD—standard deviation, *p* < 0.05—statistically significant difference, ES—effect size.

**Table 2:** Body composition: body mass (BM), body fat (BF) and android body fat (ANDR) in the aerobic group (EG1), aerobic-resistance group (EG2), and control group (CG).

	Group	Week 1 Baseline	Week 6 Intervention	Week 12 Intervention	Week 16 Follow up	*p*-Value			
		$\bar{X}$ ± SD	$\bar{X}$ ± SD	$\bar{X}$ ± SD	$\bar{X}$ ± SD	Test ANOVA (ES)	d 6-1 (ES)	d 12-1 (ES)	d 16-1 (ES)
BM [kg]	EG1	113.6 ± 16.8	111.0 ± 16.85	111.3 ± 17.47	111.4 ± 18.09	<0.001 (0.36)	<0.001 (1.07)	0.01 (0.88)	0.03 (0.71)
	EG2	107.2 ± 17.36	107.1 ± 16.41	105.2 ± 16.68	107.3 ± 17.19	0.29 (0.00)	0.50 (0.30)	0.37 (0.24)	0.32 (0.41)
	CG	109.0 ± 17.78	111.5 ± 19.09	113.7 ± 9.12	115.3 ± 19.33	0.25 (0.00)	0.67 (0.66)	0.13 (0.26)	0.22 (0.11)
	*p*-value	0.34	0.60	0.29	0.54				
ANDR [%]	EG1	48.56 ± 5.97	47.32 ± 6.09	46.87 ± 6.67	46.71 ± 5.43	0.04 (0.02)	0.05 (0.61)	0.03 (0.67)	0.02 (0.72)
	EG2	46.23 ± 6.35	45.10 ± 5.92	43.82 ± 6.41	44.70 ± 6.47	0.22 (0.01)	0.27 (0.37)	0.10 (0.58)	0.69 (0.13)
	CG	47.54 ± 6.64	48.00 ± 6.36	48.80 ± 6.85	48.52 ± 8.35	0.75 (0.00)	0.41 (0.29)	0.41 (0.29)	0.61 (0.18)
	*p*-value	0.60	0.40	0.16	0.41				
BF [kg]	EG1	42.48 ± 11.05	41.01 ± 11.12	40.96 ± 11.56	40.67 ± 11.26	0.01 (0.01)	<0.001 (1.05)	0.01 (0.77)	0.01 (0.84)
	EG2	39.52 ± 10.95	39.10 ± 10.34	37.32 ± 9.73	37.28 ± 10.31	<0.001 (0.01)	0.02 (0.92)	0.01 (1.07)	<0.001 (1.36)
	CG	39.82 ± 10.00	41.24 ± 11.67	42.77 ± 11.67	43.92 ± 11.68	0.33 (0.00)	0.76 (0.11)	0.10 (−0.62)	0.90 (−0.05)
	p-value	0.72	0.86	0.44	0.37				

d 6-1, d 12-1, d 16-1—differences in results after 6 and 12 weeks of training, respectively, and after 4 weeks of follow-up compared to measurements obtained before training sessions, $\bar{X}$—mean, SD—standard deviation, *p* < 0.05—statistically significant difference, ES—effect size.

**Table 3:** Concentrations of quantitative insulin sensitivity check index (QUICKI), nonHDL-C and HDL-C cholesterol in the participants’ blood in the aerobic group (EG1), aerobic-resistance group (EG2), and control group (CG).

	Group	Week 1 Baseline	Week 6 Intervention	Week 12 Intervention	Week 16 Follow up	*p*-Value			
		$\bar{X}$ ± SD	$\bar{X}$ ± SD	$\bar{X}$ ± SD	$\bar{X}$ ± SD	Test ANOVA (ES)	d 6-1 (ES)	d 12-1 (ES)	d 16-1 (ES)
QUICKI	EG1	0.32 ± 0.03	0.33 ± 0.04	0.32 ± 0.03	0.34 ± 0.03	0.02 (0.03)	0.04 (−0.78)	0.04 (−0.77)	0.01 (−0.98)
	EG2	0.32 ± 0.03	0.315 ± 0.04	0.34 ± 0.03	0.34 ± 0.03	<0.001 (0.05)	0.04 (0.77)	0.24 (−0.40)	0.09 (−0.61)
	CG	0.31 ± 0.04	0.31 ± 0.02	0.31 ± 0.03	0.32 ± 0.04	0.13 (0.08)	0.19 (0.34)	0.21 (0.49)	0.50 (0.30)
	*p*-value	0.60	0.07	0.06	0.12				
nonHDL-C [mmol/l]	EG1	3.88 ± 1.34	3.53 ± 1.34	3.40 ± 1.28	4.01 ± 1.55	0.05 (0.02)	0.14 (0.51)	0.10 (0.58)	0.83 (−0.07)
	EG2	4.05 ± 0.79	3.80 ± 0.64	3.92 ± 0.87	4.06 ± 0.76	0.70 (0.01)	0.19 (0.45)	0.37 (0.30)	0.60 (0.17)
	CG	4.56 ± 0.80	4.54 ± 0.97	4.58 ± 1.10	4.50 ± 0.97	0.71 (0.01)	0.62 (0.06)	0.53 (0.14)	0.49 (0.14)
	*p*-value	0.17	0.03 *	0.03 **	0.52				
HDL-C [mmol/l]	EG1	1.20 ± 0.29	1.15 ± 0.19	1.22 ± 0.36	1.27 ± 0.34	0.07 (0.04)	0.03 (0.83)	0.78 (−0.09)	0.75 (−0.11)
	EG2	1.09 ± 0.22	1.14 ± 0.26	1.15 ± 0.26	1.18 ± 0.32	0.57 (0.01)	0.59 (−0.18)	0.63 (−0.16)	0.30 (−0.35)
	CG	1.15 ± 0.19	1.16 ± 0.19	1.13 ± 0.23	1.18 ± 0.27	0.38 (0.03)	0.86 (0.06)	0.70 (0.14)	0.15 (−0.53)
	*p*-value	0.47	0.96	0.98	0.72				

* post-hoc: EG1-CG: *p* = 0.03; ** post-hoc: EG1-CG: *p* = 0.04, QUICKI—quantitative insulin sensitivity check index, nonHDL-C—non-high-density lipoprotein, HDL-C—high-density lipoprotein, d 6-1, d 12-1, d 16-1—differences in results after 6 and 12 weeks of training, respectively, and after 4 weeks of follow-up compared to measurements obtained before training sessions, $\bar{X}$—mean, SD—standard deviation, *p* < 0.05—statistically significant difference, ES—effect size.

**Table 4:** Concentrations of leptin (LEP) and omentin (OMEN) in participants’ blood plasma in the aerobic group (EG1), aerobic-resistance group (EG2), and control group (CG).

	Group	Week 1 Baseline	Week 6 Intervention	Week 12 Intervention	Week 16 Follow up		*p*-Value		
		$\bar{X}$ ± SD	$\bar{X}$ ± SD	$\bar{X}$ ± SD	$\bar{X}$ ± SD	Test ANOVA (ES)	d 6-1 (ES)	d 12-1 (ES)	d 16-1 (ES)
LEP [ng/ml]	EG1	4.96 ± 4.63	13.41 ± 8.35	4.80 ± 4.58	15.03 ± 9.6	<0.001 (0.32)	0.01 (0.70)	0.92 (0.08)	<0.001 (0.76)
	EG2	9.89 ± 5.93±	7.93 ± 4.48	7.36 ± 4.06	7.53 ± 5.14	0.01 (0.06)	0.01 (0.70)	0.02 (0.67)	0.05 (0.56)
	CG	7.54 ± 5.77	16.18 ± 11.53	8.35 ± 6.07	8.54 ± 6.15	0.01 (0.21)	0.03 (0.52)	0.79 (0.02)	0.87 (0.20)
	*p*-value	0.06	0.03 *	0.16	0.03 **				
OMEN [ng/ml]	EG1	276.03 ± 108.72	316.24 ± 132.97	334.76 ± 153.53	339.05 ± 123.09	0.53 (0.04)	0.33 (0.13)	0.32 (0.29)	0.19 (0.34)
	EG2	303.93 ± 248.13	362.77 ± 262.40	345.86 ± 291.12	282.00 ± 248.14	0.24 (0.03)	0.14 (−0.51)	0.26 (−0.38)	0.54 (0.20)
	CG	340.92 ± 176.91	322.88 ± 177.32	381.32 ± 240.40	269.07 ± 172.61	0.92 (0.01)	0.83 (0.08)	0.90 (0.14)	0.61 (0.27)
	*p*-value	0.65	0.79	0.86	0.58				

* post-hoc: EG2-CG: *p* = 0.03; ** post-hoc: EG1-EG2: *p* = 0.04; d 6-1, d 12-1, d 16-1—differences in results after 6 and 12 weeks of training, respectively, and after 4 weeks of follow-up compared to measurements obtained before training sessions, $\bar{X}$—mean, SD—standard deviation, *p* < 0.05—statistically significant difference, ES—effect size.

**Table 5:** The value of the Pearson correlation for variables in the aerobic group (EG1), aerobic-resistance group (EG2), and control group (CG).

	MET ^1^ [min/week]	Energy Value of Diet ^1^ [kJ/day]	BM ^1^ [kg]	BF ^1^ [kg]	ANDR ^1^ [%]	QUICKI ^1^	nonHDL-C ^1^ [mmol/l]	HDL-C ^1^ [mmol/l]	LEP ^1^ [ng/ml]	OMEN ^1^ [ng/ml]
LEP EG1 [ng/ml]	−0.37 *	0.28 *	0.28 *	0.39 *	0.39 *	−0.45 *	0.50 *	−0.43 *	1.00	0.16
LEP EG2 [ng/ml]	−0.21	0.09	0.73 *	0.88 *	0.87 *	−0.53 *	−0.28	−0.09	1.00	0.03
LEP CG [ng/ml]	−0.25	0.26	0.42 *	0.51 *	0.47 *	−0.20	−0.09	−0.02	1.00	−0.01
OMEN EG1 [ng/ml]	−0.14	0.14	0.09	0.14	0.13	−0.07	0.25	−0.06	0.16	1.00
OMEN EG2 [ng/ml]	0.27	0.32	0.19	0.08	0.18	−0.27	−0.39 *	0.15	0.03	1.00
OMEN CG [ng/ml]	−0.15	−0.03	−0.39 *	−0.36	−0.25	0.27	−0.34	0.31 *	−0.01	1.00
QUICKI EG1	0.29 *	−0.35 *	−0.67 *	−0.61 *	−0.56 *	1.00	−0.30	0.72 *	−0.45 *	−0.07
QUICKI EG2	0.20	−0.22	−0.64 *	−0.55 *	−0.44 *	1.00	0.44	0.27	−0.53 *	−0.27
QUICKI CG	0.10	0.04	−0.41 *	−0.33*	−0.25	1.00	−0.24	0.28*	−0.20	0.27

*—statistically significant value *p* < 0.05; LEP EG1—concentrations of leptin in EG1 taken from the four timepoints; LEP EG2—concentrations of leptin in EG2 taken from the four timepoints; LEP CG—concentrations of leptin in CG taken from the four timepoints; OMEN EG1—concentrations of omentin in EG1 taken from the four timepoints; OMEN EG2—concentrations of omentin in EG2 taken from the four timepoints; OMEN CG—concentrations of omentin CG taken from the four timepoints, MET—metabolic equivalent of task, BM—body mass, BF—body fat, ANDR—android body fat, QUICKI—quantitative insulin sensitivity check index, nonHDL-C—non-high-density lipoprotein, HDL-C—high-density lipoprotein, ^1^—taken from the four timepoints, corresponding to the group and measurement week in column 1.

**Table 6:** Parameters of multiple regression model of the leptin (LEP) dependent variable.

Dependent Variable	Parameter Assessment	Standard Error	t Value	*p*-Value
Free parameter	5.98	6.42	0.93	0.35
BF [kg]	0.31	0.06	4.98	<0.001
QUICKI	−28.05	14.88	−1.89	0.06

Free parameter—intercept, BF—body fat, QUICKI—quantitative insulin sensitivity check index.

## Data Availability

Data are available on request from the corresponding author.

## Supplementary Materials

The following supporting information can be downloaded at: https://www.mdpi.com/article/10.3390/jcm12082822/s1, Figure S1: A comprehensive strategy for aerobic program intended for a group engaged in aerobic activities (EG1). Table S1: A comprehensive strategy for a combined aerobic and resistance training program intended for a group engaged in aerobic-resistance activities (EG2). Table S2: The progressions of loads [kg] in selected resistance exercises during the intervention and follow-up in comparison to baseline in the aerobic-resistance group EG2. References [44,72] are cited in Supplementary Materials.
