# Supplementary material for: Impact of Two Types of Exercise Interventions on Leptin and Omentin Concentrations and Indicators of Lipid and Carbohydrate Metabolism in Males with Metabolic Syndrome
Source: J Clin Med. 2023 Apr 12;12(8):2822. doi: 10.3390/jcm12082822 (PMC10144374; doi:10.3390/jcm12082822)
Supplement: Supplementary file 1 [file jcm-12-02822-s001.zip › jcm-2294435-supplementary.pdf]

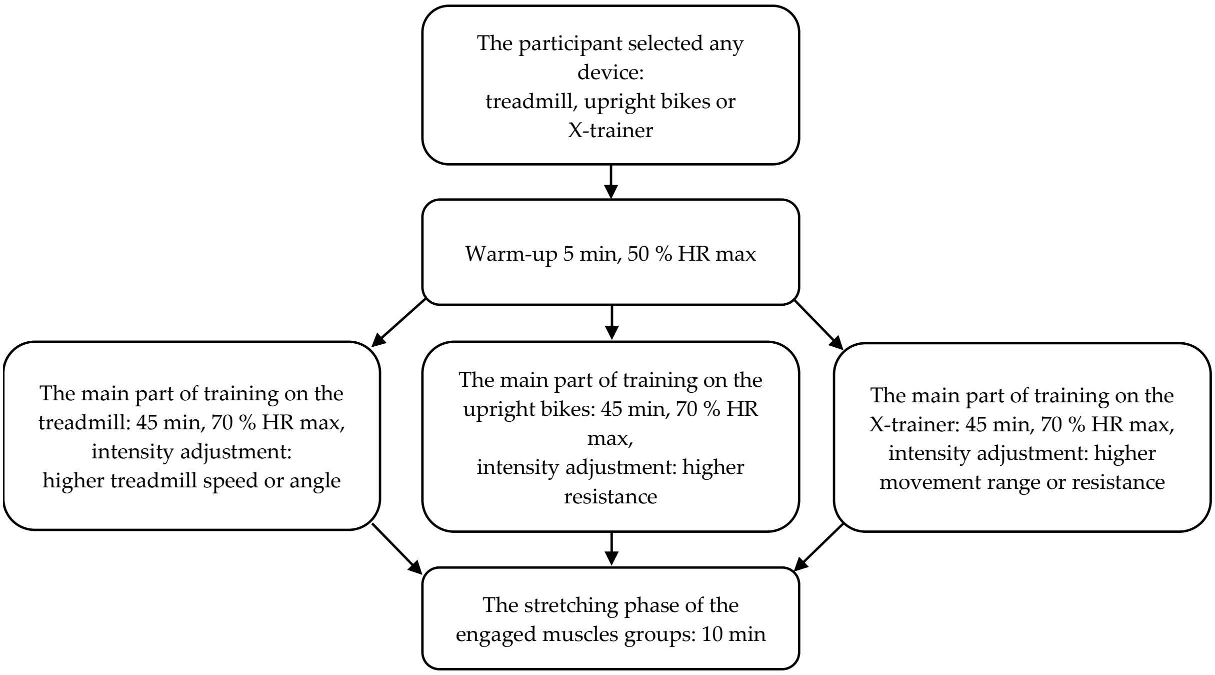

HR max – maximal heart rate

Figure S1. A comprehensive strategy for aerobic program intended for a group engaged in aerobic activities (EG1).

Table S1. A comprehensive strategy for a combined aerobic and resistance training program intended for a group engaged in aerobic-resistance activities (EG2).

	**Trainings 1 - 3**	**Trainings 4 - 6**	**Trainings 6 <**
Type of training	Whole body training	Training of antagonistic parts	Training of antagonistic parts
Volume of resistance training [exercises x series x repetitions]	3 x 4 x 15	6 x 3 x 12	9 x 3 x 12
Intensity of resistance training [% 1 RM]	50	70	70
Breaks between series [min]	2	1.5	1
Duration of resistance training [min]	30	35	40
Duration of aerobic training [min]	20	15	10
Intensity of aerobic	50	70	70

training [% HR max]			
Specialised exercises	One arm row with dumbbell<br>Supported push-ups (Smith machine)<br>Supported sit-ups (with bar)<br>Front support (plank)	Standing dumbbell press<br>Barbell bench press<br>Reverse grip lat pulldown<br>Hip thrust lying<br>Bent dumbbell row	Dumbbell deadlift<br>Cable tricep extension<br>Standing dumbbell curl

HR max – maximal heart rate, 1RM – one repetition maximum.

Table S2. The progressions of loads [kg] in selected resistance exercises during the intervention and follow-up in comparison to baseline in the aerobic-resistance group EG2.

Time of Observation	Barbell Bench Press [kg]	Lat Pull Down [kg]	Dumbbell Squat [kg]
Baseline	63.36 ± 12.92	11.58 ± 2.56	44.51 ± 8.26
After 6 weeks of intervention	72.78 ± 14.77	13.15 ± 2.84	51.77 ± 9.84
After 12 weeks of intervention	76.65 ± 15.04	14.89 ± 2.64	56.78 ± 9.77
After 16 weeks, follow up period	79.32 ± 17.29	14.91 ± 2.02	57.74 ± 10.78
p- value	0.00	0.00	0.00

p-value – ANOVA test.

The course of 1RM:

The examined participants underwent the 1 RM test before the examination, and after 6, 12, and 16 weeks.

The personal coach carried out the warm-up on the treadmill (Technogym New Excite Run Now 500, Cesena, Italy) for 5 min at 60% HR. The subjects warmed-up in two series of 10 repetitions using about 50% of their 1 RM estimated load before the beginning of the test protocol.

After 5 min break, the subjects were instructed to do the selected test exercise till the lack of possibility to continue the series of exercise maintaining the proper technique (failure).

For the 1RM bench press test, the subjects were instructed to maintain 5-point body contact (i.e., head, back, and hips with the bench, and both feet with the floor) during the test, the barbell had to touch the chest when lowered.

In the 1RM squat test, subjects were instructed to move from a standing position to a position of 90 degrees of flexion at the knee joints.

The pull-down test was performed on a training atlas. The repetition was passed when the subject made a full extension of the arms during the eccentric phase and touching the bar to the chest during the concentric phase.

A qualified personal coach controlled the range of motion to verify the correctness of the test

The last repetition of a series occurred when the participant could not continue to exercise maintaining the proper technique.

The obtained load and number of repetitions were converted into 1 RM based on the 1 RM calculator [1], applying the Brzycki formula [2].

1. Grgic, J.; Lazinica, B.; Schoenfeld, B.J.; Pedisic, Z. Test-Retest Reliability of the One-Repetition Maximum (1RM) Strength Assessment: a Systematic Review. Sports Med. Open. 2020, 6, 31. doi: https://10.1186/s40798-020-00260-z.
2. Brzycki, M. Strength Testing—Predicting a One-Rep Max from Reps-to-Fatigue. J. Phys. Educ. Recreat. Dance. 1993, 64, 88-90. doi: https://10.1080/07303084.1993.10606684
